# Determinants of Inadequate Empiric Antimicrobial Therapy in ICU Sepsis Patients in Al-Madinah Al-Munawwarah, Saudi Arabia: A Comparison of Artificial Neural Network and Regression Analysis

**DOI:** 10.3390/antibiotics12081305

**Published:** 2023-08-10

**Authors:** Ahmad Habeeb Hattab Dala Ali, Sabariah Noor Harun, Noordin Othman, Baharudin Ibrahim, Omer Elhag Abdulbagi, Ibrahim Abdullah, Indang Ariati Ariffin

**Affiliations:** 1 Discipline of Clinical Pharmacy, School of Pharmaceutical Sciences, Universiti Sains Malaysia (USM), Penang 11800, Malaysia; 2 Department of Pharmacy Practice, College of Pharmacy, AlMaarefa University, Dariyah, Riyadh 13713, Saudi Arabia; 3 Department of Clinical and Hospital Pharmacy, College of Pharmacy, Taibah University, Al-Madinah Al-Munawwarah 42353, Saudi Arabia; 4 School of Pharmacy, Management and Science University, University Drive, Off Persiaran Olahraga, Shah Alam 40100, Malaysia; 5 Faculty of Pharmacy, University of Malaya, Wilayah Persekutuan Kuala Lumpur 50603, Malaysia; 6 Department of Surgery, King Salman Medical City, Medina 42316, Saudi Arabia; 7 Research Management Centre, Management and Science University, University Drive, Off Persiaran Olahraga, Section 13, Shah Alam 40100, Malaysia

**Keywords:** sepsis, septic shock, empiric antimicrobials, determinants, neural networks, artificial intelligence

## Abstract

In the management of sepsis, providing adequate empiric antimicrobial therapy is one of the most important pillars of sepsis management. Therefore, it is important to evaluate the adequacy of empiric antimicrobial therapy (EAMT) in sepsis patients admitted to intensive care units (ICU) and to identify the determinants of inadequate EAMT. The aim of this study was to evaluate the adequacy of empiric antimicrobial therapy in patients admitted to the ICU with sepsis or septic shock, and the determinants of inadequate EAMT. The data of patients admitted to the ICU units due to sepsis or septic shock in two tertiary healthcare facilities in Al-Madinah Al-Munawwarah were retrospectively reviewed. The current study used logistic regression analysis and artificial neural network (ANN) analysis to identify determinants of inadequate empiric antimicrobial therapy, and evaluated the performance of these two approaches in predicting the inadequacy of EAMT. The findings of this study showed that fifty-three per cent of patients received inadequate EAMT. Determinants for inadequate EAMT were APACHE II score, multidrug-resistance organism (MDRO) infections, surgical history (lower limb amputation), and comorbidity (coronary artery disease). ANN performed as well as or better than logistic regression in predicating inadequate EAMT, as the receiver operating characteristic area under the curve (ROC-AUC) of the ANN model was higher when compared with the logistic regression model (LRM): 0.895 vs. 0.854. In addition, the ANN model performed better than LRM in predicting inadequate EAMT in terms of classification accuracy. In addition, ANN analysis revealed that the most important determinants of EAMT adequacy were the APACHE II score and MDRO. In conclusion, more than half of the patients received inadequate EAMT. Determinants of inadequate EAMT were APACHE II score, MDRO infections, comorbidity, and surgical history. This provides valuable inputs to improve the prescription of empiric antimicrobials in Saudi Arabia going forward. In addition, our study demonstrated the potential utility of applying artificial neural network analysis in the prediction of outcomes in healthcare research.

## 1. Introduction

Sepsis is considered a medical emergency that involves a systemic immunological response due to an infection, resulting in end-stage organ malfunction and death [1]. It has been reported as one of the major causes of morbidity and mortality in intensive care units (ICU) and is considered the most common death cause in non-coronary intensive care units, with a mortality rate of approximately 20–25% [2,3].

**Figure 1:**
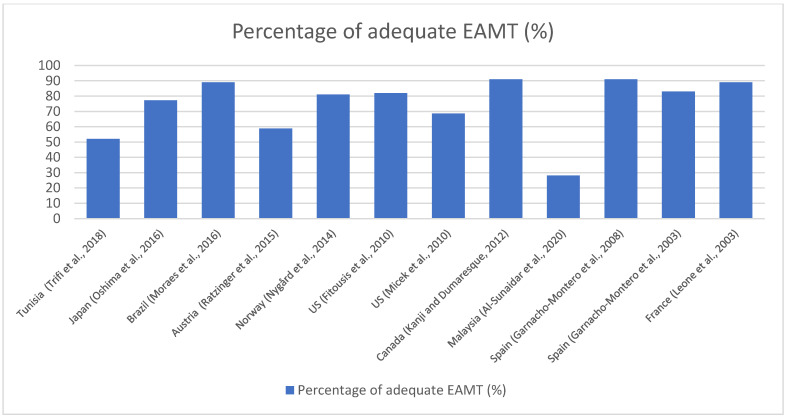
Percentages of adequate empiric antimicrobial therapy in sepsis patients as reported in the literature [5,6,7,8,9,11,12,14,15,16,17,18].

In managing sepsis, therapies are provided to manage the basic elements of sepsis, including the eradication of the underlying infection, the prevention and treatment of organ dysfunction, and host response [2]. Therefore, initial resuscitation through restoring the tissue perfusion and early empiric antimicrobial therapy are rendered among the pillars of sepsis management [4]. Several studies have demonstrated the significant association of inadequate EAMT with increased mortality in ICU sepsis patients [5,6,7,8]. Reasons for the inadequacy of EAMT include the resistance of the isolated microorganism to the provided empiric agents, the selection of empiric agents not according to the clinical practice guidelines, the dose of empiric agents not in accordance with the recommended dose by the guidelines, and delays in providing adequate EAMT [5,9]. This due to the fact that adequate empiric antimicrobial therapy is optimally selected to cover the causative pathogen and should also be provided at the optimal time; it also involves the avoidance of overdosing or under-dosing, i.e., for EAMT to be considered adequate it should include the correct agent and the right dose, and be administered in a timely manner [10]. The adequacy of EAMT provided to different sepsis patient populations, including ICU sepsis patients, surgical ICU sepsis patients, and sepsis patients admitted to medical and surgical wards, has been described by several studies worldwide [5,7,8,9,11,12]. Sepsis definition has evolved over the past decades, and therefore there are inconsistent applications of sepsis definitions which can potentially result in a variability of sepsis epidemiology in the literature [1,13]. In addition, the percentages of adequate empiric antimicrobial therapy reported in the literature varied (Figure 1). This can be due to the lack of a consistent definition of sepsis and adequate EAMT used in these studies [5,6,7,9,12,14,15,16,17,18]. There have been several studies conducted among ICU sepsis patients in Saudi Arabia. However, most of these studies focused on the spectrum and antimicrobial sensitivity of the bacteria pathogens in neonatal sepsis [19], the impact of empiric antimicrobial therapy on the mortality of septic shock patients with liver cirrhosis [20], and pediatric ICU patients [21]. In addition, there is a lack of evidence on the adequacy of empiric antimicrobials provided to ICU sepsis patients and their determinants in Middle Eastern countries [22].

Artificial Neural Network (ANN) possesses the ability to collect samples’ information, learn, make generalizations, and make decisions regarding samples that they have not seen before (i.e., test sets) by using previously learnt information (i.e., training sets) [23]. In addition, ANN models are considered a flexible, complex, non-linear modeling technique that possesses properties which are not found in other modeling systems, including a powerful performance in dealing patterns that are incomplete or noisy, and high fault tolerance [24]. Therefore, the artificial neural network is very useful when dealing with complex relationships and implicit interactions in the data [25].

The prediction of outcomes is an important aspect of healthcare research in the discipline of critical care, which demonstrates the potential utility of ANN analysis in such research disciplines [24]. In recent years, some researchers have used ANN and compared its performance with regression analysis. For instance, Fardamal and colleagues assessed the utility of ANN in the prediction of survival in breast cancer patients, and they reported that ANN was found to give a better performance in the prediction of survival when compared with logistic regression models (LRM), with AUC of 0.834 and 0.744, respectively [26]. Furthermore, ANN was found to possess a better performance when compared with LRM in the prediction of lower back pain [27]. However, there have been no studies utilizing such analysis (i.e., artificial neural network) in predicting the determinants of EAMT adequacy. Therefore, the aim of this study was to assess the adequacy of EAMT and the determinant of inadequate EAMT in ICU sepsis patients at Al-Madinah Al-Munawwarah. This study also utilized ANN analysis and compared its performance with traditional regression models, i.e., logistic regression models (LRM), in predicting the outcome of interest.

## 2. Results

In the current study, we screened a total of 1477 records of patients admitted to two centers (Medina General Hospital—King Salman Medical City, and Miqat General Hospital) from July 2019 to June 2021. A total of 292 patients were diagnosed with sepsis/septic shock, which makes the prevalence of sepsis/septic shock 19.8%. However, only 253 patients met our inclusion criteria and therefore were included in the study.

The mean age of the patients was 67.6 ± 15.1. Of the 253 patients, 61.7% of them were male and only 38.3% of them were female. Only 5.1% of patients were Hajj and Umrah visitors, while the remaining 94.9% were either citizens or residents. More than half of the patients (55.3%) were diagnosed with septic shock, while 44.7% of them were diagnosed with sepsis. The mean number of comorbidities was 2.3 ± 0.9, and the most frequently reported comorbidities were hypertension and diabetes mellitus, (84.6%) and (84.4%), respectively. More than half of the patients (56.9%) had a moderate GCS score (9–12). The mean score of the APACHE II scores was 27.1 ± 8.4. The majority of patients (70%) were admitted to the ICU from the emergency departments. The majority of patients (70.4%) developed sepsis/septic shock due to community-acquired infections. The most frequently reported source of infection was respiratory tract infections (45.8%). Thirty-four per cent of patients had had previous surgery, of which 20.6% had had the surgery within one week or during the ICU admission; lower limb amputation was the most common type of surgery. The mean number of organ failure incidence among our cohort was 3.5 ± 1.78. Almost thirty one percent of causative organisms (30.8%) were classified as multiple drug resistant microorganism (MRDO) infections (Table 1).

**Figure 2:**
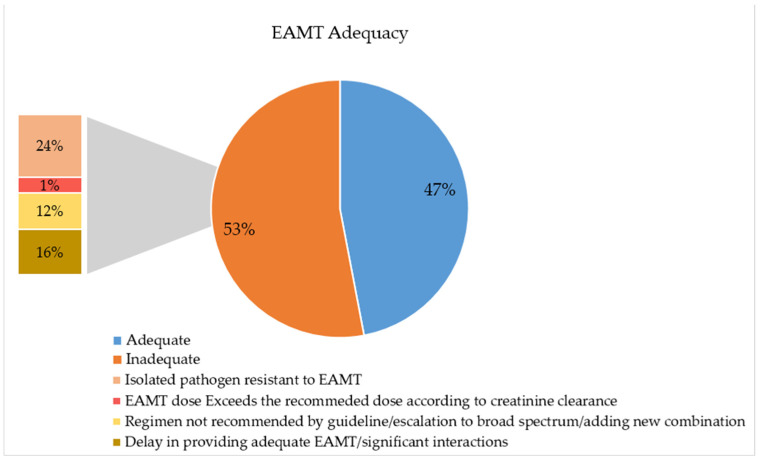
Empiric Antimicrobial Therapy (EAMT)’s adequacy.

With regard to the adequacy of empiric antimicrobial therapy used in our cohort, more than half of the provided EAMT (53%) were found to be inadequate. The most common cause of inadequacy was the resistance of the isolated microorganism to the EAMT (24%), followed by a delay in providing the EAMT regimen (16%) (Figure 2).

According to the univariate regression analysis, potential determinants (i.e., risk factors) significantly associated with empiric antimicrobial therapy were age, diagnosis (septic shock), site which patients were admitted to the ICU from, surgical history, surgical intervention within the past week, type of surgery (lower limb amputation), day 1 GCS score, number of co-morbidities, type of co-morbidities (hypertension and coronary artery disease), need for mechanical ventilation, number of incidences of organ failure, type of organ failure (acute kidney injury, liver failure, cardiac failure, DIC, respiratory failure, and central nervous system insult), day 1 APACHE II score, type of infection (respiratory tract infection and soft tissue/skin infection), and MDRO infection Table S1.

However, the multivariate regression analysis revealed that patients who had coronary artery disease as a comorbidity significantly increased the odds of inadequate EAMT by 3.128 (OR 3.128, 95% CI 1.016–9.629, *p* = 0.047), with a higher APACHE II score (OR 1.087, 95% CI 1.010–1.170, *p* = 0.026), i.e., each one-unit increase in the APACHE score increased the odds of inadequate EAMT by 1.087, and MDRO infection was found to significantly increase the odds of inadequate EAMT by 7.318 (OR 7.318, 95% CI 2.839–18.864, *p* = <0.001). In contrast, patients who had surgical intervention (lower limb amputation) were found to be less likely to have inadequate EAMT, with an odds ratio of 0.019 (OR 0.019, 95% CI 0.025–0.478, *p* = 0.003); see Table 2.

The classification accuracy of the ANN outperformed the logistic regression model, as shown by (Table 3) the ANN training and testing sets’ accuracy for inadequate EAMT; 80.0% and 82.0%, respectively, while the logistic regression model was able to correctly classify inadequate EAMT by 76.1%. In addition, the overall percentage correct of prediction was found to be higher in the ANN model when compared with the logistic regression model (Table 3).

**Figure 3:**
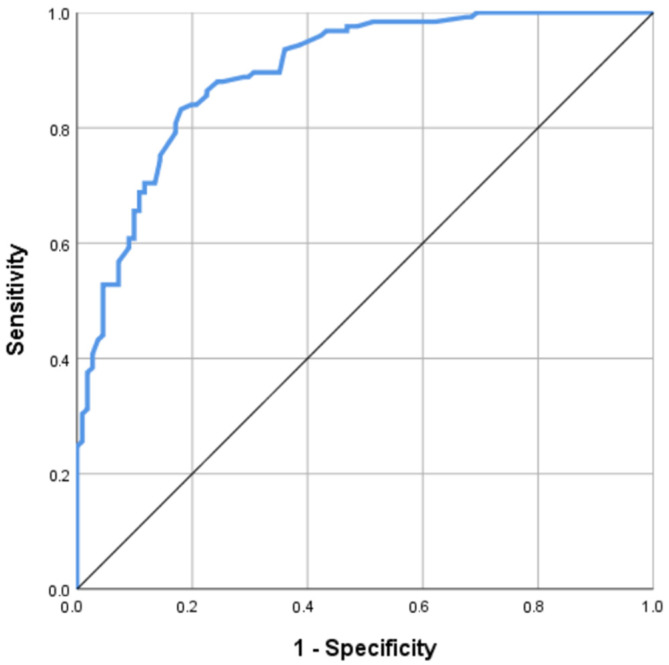
Receiver Operating Characteristic curves for the prediction of inadequate EAMT generated from the artificial neural network model. Note: AUC = 0.895.

**Figure 4:**
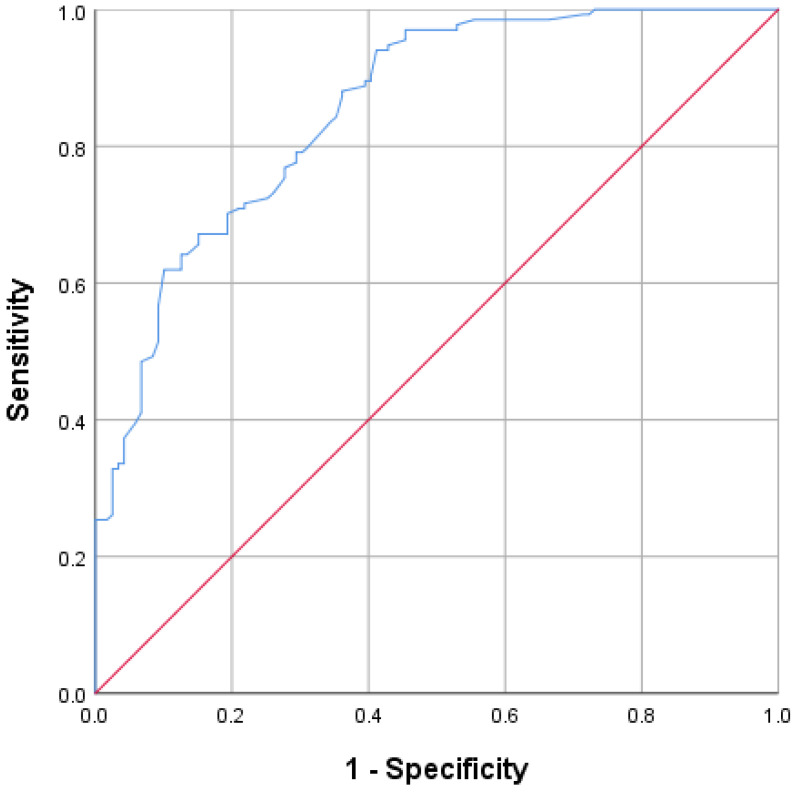
Receiver Operating Characteristic curves for the prediction of inadequate EAMT generated from the logistic regression model. Note: AUC = 0.854.

The Receiver Operating Characteristic (ROC) curve for inadequate EAMT for the ANN and logistic regression models were developed. The ANN model’s area under the curve (ROC-AUC) based on both training and testing samples was 0.895, indicating a very good classification accuracy rate; see Figure 3. Also, when compared with the ROC-AUC of the regression model, the ANN had a higher ROC-AUC: 0.895 vs. 0.845 (Figure 3 and Figure 4).

Table 4, below, indicates the determinants’ importance in the prediction of the inadequate EAMT. The APACHE II scores were found to be the most important determinant of inadequate EAMT (importance: 0.489; normalized importance: 100%), followed by MDRO infection (importance: 0.214; normalized importance: 43.9%), surgical history (importance: 0.160; normalized importance: 32.7%), and coronary artery disease (importance: 0.137; normalized importance: 28.0%).

## 3. Discussion

The percentage of inadequate empiric antimicrobial therapy in our cohort was 53%. Numerous studies in the literature that investigated the percentages of inadequate empiric antimicrobial therapy have shown inconsistent findings. These studies reported that inadequate EAMT was provided to 18.0%, 18.6%, and 11.0% of sepsis patients admitted to the ICU in United States [11], Norway [12], and France [15], respectively. Much higher percentages were reported in other countries; for example, in Tunisia (48.0%) [8], and 71.9% in Malaysia [5] and 41.1% in Austria [7]. This variation could be due to the inconsistency of the definition of EAMT’s adequacy used by these studies, as most studies defined the adequacy of EAMT according to the culture and sensitivity results, while several studies took into account the timing, dose, and compliance with the guideline recommendations when assessing the EAMT adequacy [28]. Furthermore, variations in the settings, infectious agents, and diseases could potentially impart a variation in the reported percentages of inadequate EAMT in the literature [22,28]. Such variations were also reported through a meta-analysis conducted by Kristel and colleagues, which reported that the percentages of inappropriate empiric antimicrobial therapy showed an enormous range, from 14.1 to 78.9%. However, out of the 27 studies included in the meta-analysis, 13 studies reported a percentage of inappropriate EAMT of 50% or more [28].

Several studies have indicated that providing inadequate empiric antimicrobial therapy can significantly result in poor clinical outcomes in sepsis patients [5,6,7,8]. This shows the importance of assessing the adequacy of EAMT and its determinants, as the decision of the selection of empiric antimicrobial therapy is very challenging, which indicates the importance of identifying the determinants of inadequate EAMT, which subsequently contributes significantly to the improvement of the rational use of antimicrobials [29]. With this in mind, according to the multivariate regression analysis of our study data, it revealed that factors significantly associated with inadequate EAMT were coronary artery disease as a comorbidity, a higher APACHE II score, and MDRO infection. On the other hand, patients with a surgical history of lower limb amputation were less likely to receive inadequate EAMT.

Within a similar context, similar results were obtained by other studies in the literature assessing the determinants of inadequate EAMT. For instance, a study conducted by Yokota et al. (2014) identified that the presence of polymicrobial infection and a higher APACHE II score were independently associated with inadequate antimicrobial therapy among patients with severe sepsis and septic shock. This can be explained as an underestimation of the patient’s severity of illness, i.e., neglecting the patient’s severity of illness can affect the decision to use optimal antimicrobial therapy [30]. Also, this could be due to the complexity of using severity of illness scoring systems, and the prolonged time needed for calculating the scores can include difficulties in using such scoring systems [31]. In addition, coronary artery disease (CAD) was associated with the increased likelihood of receiving inadequate EAMT. In this regard, Tirifi and colleagues (2018) also identified chronic co-morbidities (chronic respiratory failure) to be a significantly associated with the appropriateness of EAMT [8]. This also can be considered an implication of not including comorbidities in the APACHE II score. Therefore, it can be reflected as an under-estimation of patients’ severity of illness and can subsequently result in providing inadequate therapy [30,32,33]. In addition, one of the important strategies to ensure the adequacy of the provided therapy is to identify patients at high risk, which includes patients with nosocomial infection and co-morbidities [34].

The association of MDRO with inadequate empiric antimicrobial therapy was frequently identified by studies in the literature. For instance, according to a meta-analysis carried out by Carrara and colleagues (2018), multidrug resistant organisms were identified as being significantly associated with inappropriate empiric antibiotic treatment and with poor clinical outcomes [35]. MDRO infections remain one of the most commonly reported determinants of inadequate EAMT in both community- and hospital-acquired infections, as they have been reported to be one of the independent risk factors of inadequate EAMT in different critically ill patient populations, including patients with bacteremia [36] and patients admitted to the hospital due to urinary tract infections, pneumonia, and sepsis [37]. Moreover, risk factors for inappropriate empiric antimicrobial therapy were found to be similar to the identified risk factors for fluoroquinolones in a study including a hospitalized patient with urinary tract infections [38]. This shows the importance of the identification of independently associated modifiable and non-modifiable risk factors (i.e., predictors) of MDRO infections in sepsis patients, and the development of a scoring system for the early determination of the likelihood of patients to have an MDRO infection. In addition, taking into account the patients’ severity of illness, co-morbidities, and local epidemiologic data were important for developing rapid diagnostic tools to guide the clinicians in selecting the most appropriate and efficient antimicrobial therapy [39].

In our study, patients with a history of surgical procedures (limb amputation) were found to be significantly less likely to receive inadequate empiric antimicrobial therapy. Fitousis and colleagues reported in their study that appropriate empiric antimicrobial therapy was provided to 82% of patients admitted due to surgical sepsis, which reflected a successful implementation of the antimicrobial guidelines [11]. This can also reflect the adherence to the Saudi Ministry of Health National Antimicrobial Therapy Guidelines for Community- and Hospital-Acquired Infections in Adults recommendations [40]. This was also shown by a large, prospective study conducted in 150 intensive care units, which also reported that surgical intensive units were more likely to comply with the surviving sepsis campaign’s recommendations [41]. 

Compared with a traditional logistic regression model (LRM), the artificial neural network (ANN) model had a higher classification accuracy for inadequate EAMT compared with LRM, and the ROC-AUC of the ANN model was higher than LRM’s. Therefore, the ANN model performed as well as or better than the LRM model in predicting inadequate EAMT in sepsis patients. Similar results were reported by several studies that utilized ANN in the prediction of outcomes. The ROC-AUCs of ANN vs. LRM were reported to be 0.77 vs. 0.72, 0.75 vs. 0.72, and 0.97 vs. 0.95 in predicting lower-back-pain progression, ICU mortality, and thyroid disease diagnosis, respectively [25,27,42]. Given that the less error prediction model is considered a healthcare researcher’s ultimate goal, the utilization of an artificial neural network can potentially provide new opportunities to obtain predictions with enhanced accuracy [25]. This can explain the increasing trends of the utilization of ANN models making complex medical decisions and the prediction of outcomes in patients with various diseases [43]. 

According to the artificial neural network analysis, APACHE II score and MDRO infection were found to be the most important determinants of EAMT adequacy. To our knowledge, our study is the first of its kind to use an artificial neural network model to predict the EAMT adequacy in sepsis patients. Therefore, there is a lack of literature to compare. However, it is well established that the underestimation of a patient’s severity of illness subsequently affects the adequacy of EAMT and outcomes in sepsis patients, and this can explain why it is crucial to aim for the quick identification of the source of infection, and the fast administration of adequate EAMT when managing patients with sepsis [12,30]. In addition, empiric antimicrobial therapy should be carried out according to the patient’s risk factors, which include the location of the acquisition of infection, the site of the infection, the severity of the illness, the immune status, and the local antibiogram [44].

Evidently, multidrug-resistant organism infections were identified as being significantly associated with inappropriate empiric antibiotic treatment and with poor clinical outcomes [35]. Furthermore, according to a 4-year prospective study conducted by Corcione and colleagues, the introduction of empiric antimicrobial therapy manuals resulted in a decrease in the trends of antimicrobial use and MDRO blood stream infections [45]. This also shows that providing adequate empiric antimicrobial therapy will also lead to a reduction in the antimicrobial resistance, which shows the importance of determining the adequacy of EAMT and the determinants of inadequate EAMT.

Given that, the prediction of outcomes is rendered an important aspect of healthcare research in the discipline of critical care [24]. Our study demonstrated that artificial neural network analysis outperformed the regression analysis, i.e., having a higher accuracy and higher ROC-AUC. This supports the usefulness of artificial neural network models in the prediction of outcomes of interest and the potential utility of the application of deep learning and artificial intelligence methodologies in the prediction of healthcare research outcomes, potentially augmenting healthcare professionals’ capabilities [46].

This study has many strengths, which include being, to our knowledge, the first study that assesses the adequacy of empiric antimicrobial therapy in ICU sepsis patients and its predictors in Saudi Arabia. It is very challenging to make appropriate selections of adequate empiric antimicrobial therapy regimens. Therefore, providing inputs regarding inadequate antimicrobial therapy and its determinants contributes significantly to the improvement of the rational use of antimicrobials [29]. Thus, our study provides a valuable input that can be used by healthcare providers and healthcare institutes to improve empiric antimicrobial therapy prescription in the era of limited and lack of availability of new antimicrobial agents that can be used to overcome new resistant microorganisms [47]. Also, our study is the first of its kind that utilized deep learning and artificial intelligence methodologies in the prediction of inadequate EAMT. However, the current study also has some limitations, which includes the current study being conducted in a specific geographic region, i.e., Al-Madinah Al-Munawwarah. Therefore, the results of the current study should be used and interpreted with caution in practice settings outside Al-Madinah Al- Munawwarah. In addition, factors that can affect the pharmacokinetics of antimicrobials were not incorporated in this study. Thus, future research should include such parameters to evaluate the changes in antimicrobial pharmacokinetics in sepsis patients and their impact on the adequacy of EAMT provided to sepsis patients.

## 4. Materials and Methods

### 4.1. Study Design, Settings, and Populations

In this retrospective cohort observational study, medical records of all patients admitted to the intensive care units in Medina General Hospital and Miqat General Hospital from July 2019 to June 2021 were reviewed. Patients were included according to the following inclusion criteria: adult patients (>18 years old); admitted to the ICU; stayed at least 3 days in the ICU and not more than 14 days, and in case of multiple admissions, only the first admission was considered; and diagnosed with sepsis/septic shock according to the Sepsis—3 Definitions [48], where sepsis was defined as life-threatening organ dysfunction caused by a dysregulated host response to infection. Septic shock was defined as a subset of sepsis in which underlying circulatory and cellular/metabolic abnormalities were profound enough to substantially increase mortality. Exclusion criteria included COVID-19 patients, pregnant patients, patients with active malignancy, patients less than 18 years of age, and patients admitted to non-ICU wards.

### 4.2. Data Collection

All ICU admissions were reviewed from the intensive care unit admission/discharge record and statistics unit. Medical record numbers (MRNs) of patients diagnosed with sepsis or septic shock were collected. Then, based on the MRNs, data related to patients’ demographics, medical and surgical history, comorbidities, organ function, GCS score, APACHE II score, information related to onset and type of infection, and the provided EAMT were retrieved from the computerized medical records system (ALERT^®^).

### 4.3. Assessment of Empiric Antimicrobial Therapy (EAMT)’s Adequacy

The criteria of adequate empiric antibiotic therapy was developed and adapted after an extensive search of the literature [5,6,7,9,12,14,16,17,22,30,36,49,50,51]. The EAMT adequacy assessment criteria were reviewed by two experts (clinical pharmacy fellows) to ensure that the criteria would appropriately measure the purpose of the study (i.e., stratify EAMT into adequate or inadequate). Following that, to assess the applicability of the criteria, a set of 19 randomly selected cases diagnosed with sepsis or septic shock were evaluated by the experts. Inter-ratter agreement was evaluated using percent raw agreement and Cohen’s kappa (κ) statistic for the adequacy of EAMT. A kappa value with 95% confidence interval was reported to be 0.791 (0.415–0.919), which according to Landis and Koch [52] is considered substantial agreement. The strength of agreement is defined as kappa values less than zero correspond to no agreement, from zero to 0.2 correspond to slight agreement, from 0.21 to 0.4 correspond to fair agreement, from 0.41 to 0.6 correspond to moderate agreement, from 0.61 to 0.8 correspond to substantial agreement, and from 0.81 to 1 correspond to near-perfect agreement [52]. The following criteria were used in this study to describe adequate empiric antibiotic therapy: The isolated microorganisms were covered by at least one of the used empiric agents. The dose and frequency of the administered empiric antimicrobials were in accordance with local guidelines in Saudi Arabia and could cover the most likely causative pathogen depending on the site and source of infection [40]. Empiric antimicrobials had to be administered as soon as possible and not more than six hours, and the time of ICU admission and time of initiation of empiric agents were verified based on the patients’ records. In contrast, EAMT was considered inadequate if the empiric agents did not cover the isolated microorganisms, i.e., the isolated pathogen was not sensitive to any of the empiric agents used; the dose of empiric agents exceeded the guideline recommend dose according to the Creatinine clearance; there was escalation of empiric agents from narrow to broad or addition of other new combinations or agents used that were not recommended by the local guidelines; or any delay of EAMT or significant interactions.

### 4.4. Statistical Analysis

All statistical analyses were performed using Statistical Package for Social Sciences (SPSS, IBM Corp, Armonk, NY, USA), version 25.0 [53]. Descriptive statistics were used to describe the data; continuous data were presented as mean ± standard deviation (SD), and categorical data were expressed as numbers with percentages. The significance level was set at a *p* value of less than 0.05. To identify determinants of inadequate empiric antimicrobial therapy (EAMT), binary logistic regression analysis was used to identify statistically significant associated variables associated with inadequate EAMT. Multivariate regression analysis was used to identify the independently associated determinants of inadequate empiric antimicrobial therapy.

**Figure 5:**
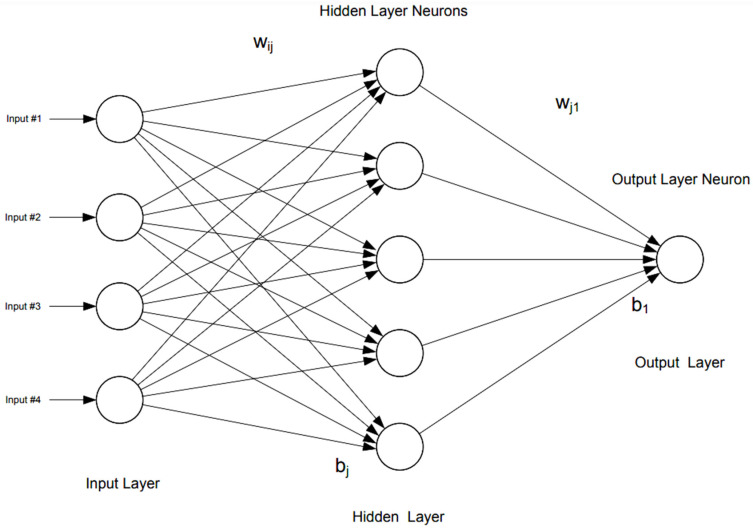
Structure and components of a typical artificial neural network (used with author’s permission) [57].

To develop ANN and to identify the importance of predictors, the multilayer perceptron function using Statistical Package for Social Sciences (SPSS), version 25.0 [53] was used to develop the ANN model of the outcome variables (i.e., Inadequate EAMT). Multiple regression analysis was used to determine the input variables for the ANN model [54,55]. For categorical outputs (i.e., Inadequate EAMT), the hyperbolic tangent activation function was used for the two hidden layers and the Softmax activation function was used for output layers. Figure 5 describes the typical components of the artificial neural network. The performance of the artificial neural network and logistic regression analysis were compared using the classification accuracy and receiver operation curve-area under the curve (ROC-AUC). In addition, independent variable importance generated from the multilayer perceptron tool was used to identify the most important variable in predicting the outcome. Table 5, below, shows the ROC-AUC values and their corresponding accuracy levels [56].

## 5. Conclusions

Providing adequate empiric antimicrobial therapy is considered one of the essential management strategies of sepsis. Our study resembles a situational analysis that describes the adequacy of EAMT provided to ICU sepsis patients and its determinants, which can potentially aid in improving antimicrobial prescription. We observed that more than half of patients received inadequate EAMT. Determinants associated with an increased likelihood of inadequate EAMT included APACHE II score, MDRO infections, and comorbidity (CAD), while a surgical history of lower limb amputation was associated with decreased odds of receiving inadequate EAMT. Furthermore, the study’s findings demonstrated that the ANN performed as well or better than logistic regression, which shows the potential utility for applying artificial neural network analysis in the prediction of outcomes in healthcare research.

## Figures and Tables

**Table 1:** ICU sepsis/septic shock patients’ characteristics.

Variables	Total n (%), or *Mean ± SD*
Age (years) **^a^**	67.6 ± 15.1
Gender **^b^**	
Male	156 (61.7)
Female	97 (38.3)
Type of residency **^b^**	
Hajj/Umrah visitor	13 (5.1)
Local/resident	240 (94.9)
Diagnosis **^b^**	
Sepsis	113 (44.7)
Septic shock	140 (55.3)
History of antibiotic use **^b^**	85 (33.6)
Admission site **^b^**	
Emergency department	177 (70.0)
Medical Wards	2 (0.8)
Surgical wards	51 (20.2)
Other institutes	23 (9.1)
Surgical history **^b^**	87 (34.4)
Time of surgery **^b^**	
Within the past week	52 (20.6)
More than 1 week–months	12 (4.7)
More than 6 months	23 (9.1)
Type of surgical history **^b^**	
Abdominal	2 (0.8)
Orthopedic	6 (2.4)
Neurosurgery	4 (1.6)
Cardiovascular	14 (5.5)
Urological	3 (1.2)
Lower limb amputation	41 (16.2)
Diabetic septic foot debridement	13 (5.1)
Fasciotomy	2 (0.8)
Malignancy	1 (0.4)
GCS score **^b^**	
Severe (≤8)	58 (22.9)
Moderate (9–12)	144 (56.9)
Minor (≥13)	51 (20.2)
Number of comorbidities **^a^**	2.3 ± 0.93
Type of comorbidities **^b^**	
Diabetes Mellitus	213 (84.2)
Hypertension	214 (84.6)
Asthma	9 (3.6)
Chronic Obstructive Pulmonary Disease	5 (2.0)
Coronary Artery Disease	60 (23.7)
Congestive Heart Disease	10 (4.0)
Chronic renal disease	11 (4.3)
Old malignancy	5 (2.0)
Liver diseases	2 (0.8)
Central Nervous System	54 (21.3)
Need for Mechanical Ventilation **^b^**	149 (58.9)
Number of organ failure **^a^**	3.47 ± 1.78
Liver failure **^b^**	190 (75.1)
Acute kidney injury **^b^**	141 (55.7)
Respiratory failure **^b^**	153 (60.5)
Central Nervous System **^b^**	147 (58.1)
Cardiac failure **^b^**	121 (47.8)
Disseminated intravascular coagulation **^b^**	110 (43.5)
Venous thromboembolism **^b^**	17 (6.7)
APACHE II score **^a^**	27.1 ± 8.4
Onset of infection **^b^**	
Hospital-acquired infection	75 (29.6)
Community-acquired infection	178 (70.4)
Source of infection **^b^**	
Respiratory tract infection	116 (45.8)
Urinary tract infection	45 (17.8)
Abdominal infection	4 (1.6)
Soft tissue/skin infection	65 (25.7)
Surgical site infection	21 (8.3)
CNS infection	1 (0.4)
Unknown	1 (0.4)
MDRO **^b^**	78 (30.8)

GCS: Glasgow Coma Scale. APACHE II: Acute Physiology and Chronic Health Evaluation. CNS: Central Nervous System. MDRO: Multiple Drug Resistant organism. **^a^**: Data were presented as mean ± SD. **^b^**: Data were presented as number (%).

**Table 2:** Univariate and multivariate regression analysis of determinants of inadequate EAMT.

Variables	Adequate	Inadequate	Variable Coefficient (B)	Crude OR (95%CI)	*p* Value	Variable Coefficient (B)	Adjusted OR (95% CI)	*p* Value
Type of surgical history **^b^**								
Lower limb amputation	32 (62.7)	9 (25.7)	−1.631	0.196 (0.089–0.431)	<0.001	−2.215	0.109 (0.025–0.478)	0.003
Type of comorbidities **^b^**								
Coronary Artery Disease	15 (12.6)	45 (33.6)	1.254	3.506 (1.831–6.710)	<0.001	1.140	3.128 (1.016–9.629)	0.047
APACHE II score **^a^**	22.76 ± 8.11	31.10 ± 6.59	0.146	1.157 (1.111–1.204)	<0.001	0.100	1.087 (1.010–1.170)	0.026
MDRO **^b^**	16 (13.4)	62 (46.3)	1.713	5.543 (2.962–10.374)	<0.001	1.990	7.318 (2.839–18.864)	<0.001

APACHE II: Acute Physiology and Chronic Health Evaluation. MDRO: Multiple Drug Resistant organism. **^a^**: Data were presented as mean ± SD. **^b^**: Data were presented as number (%).

**Table 3:** Accuracy of ANN classification in training and testing sets.

Classification				
Sample	Observed	Predicted		
		Adequate	Inadequate	Percentage Correct
Logistic regression	Adequate	86	33	72.3%
	Inadequate	32	102	76.1%
	Overall Percentage			74.3%
ANN—Training	Adequate	64	13	83.1%
	Inadequate	15	60	80.0%
	Overall Percentage	52.0%	48.0%	81.6%
ANN—Testing	Adequate	28	6	82.4%
	Inadequate	9	41	82.0%
	Overall Percentage	44.0%	56.0%	82.1%

**Table 4:** Independent variable importance in determining EAMT adequacy.

Independent Variable Importance		
	Importance	Normalized Importance
Coronary artery disease	0.137	28.0%
Surgical history	0.160	32.7%
MDRO infection	0.214	43.9%
APACHI II score	0.489	100.0%

APACHE II: Acute Physiology and Chronic Health Evaluation. MDRO: Multiple Drug Resistant organism.

**Table 5:** Discrimination quality as defined by [56].

ROC-AUC Value	Discrimination Quality
≥0.9	Outstanding
0.8–0.9	Excellent
0.7–0.8	Acceptable
0.5	No discrimination

## Data Availability

The data are not publicly available to keep confidentiality and to comply with ethical considerations.

## Supplementary Materials

The following supporting information can be downloaded at: https://www.mdpi.com/article/10.3390/antibiotics12081305/s1, Table S1: Univariate and multivariate regression analysis of variables associated with inadequate EAMT.
