# Supplementary material for: Determinants of Inadequate Empiric Antimicrobial Therapy in ICU Sepsis Patients in Al-Madinah Al-Munawwarah, Saudi Arabia: A Comparison of Artificial Neural Network and Regression Analysis
Source: Antibiotics (Basel). 2023 Aug 10;12(8):1305. doi: 10.3390/antibiotics12081305 (PMC10451895; doi:10.3390/antibiotics12081305)
Supplement: Supplementary file 1 [file antibiotics-12-01305-s001.zip › antibiotics-2378669-supplementary.pdf]

Table S1 Univariate and Multivariate regression analysis of variables associated with of inadequate EAMT

Variables	Adequate	Inadequate	Univariate *P* value	Multivariate *P* value	Adjusted OR (95% CI)
**Age**	*63.55 ±14.18*	71.28 *±15.09*	<0.001	0.608	1.008(0.978-1.040)
**Gender**					
Male	77 (64.7)	79 (59.0)	0.348		
Female	42 (35.3)	55 (41.0)	-		
**Type of residency**					
Hajj/Umrah visitor	9 (7.6)	4 (3.0)	0.112		
Local/Resident	110 (92.4)	130 (97.0)	-		
**Diagnosis**					
Sepsis	87 (73.1)	26 (19.4)	-		
Septic shock	32 (26.9)	108 (80.6)	<0.001	0.310	1.878(0.556-6.336)
**History of antibiotic use**	46 (38.7)	39 (29.1)	0.109		
**Admission site**					
Emergency department	75 (63.0)	102 (76.1)	0.024	0.493	1.589(0.423-5.963)
Medical Wards	1 (0.8)	1 (0.7)	0.933		
Surgical wards	34 (28.6)	17 (12.7)	0.002	0.478	2.306(0.230-23.143)
Other institutes	9 (7.6)	14 (10.4)	0.428		
**Surgical history**	51 (42.9)	36 (26.9)	0.008	0.339	2.312(0.415-12.896)
**Time of surgery**					
Within the past week	32 (62.7)	20 (55.6)	0.020	0.682	0.656(0.087-4.946)
More than 1 week – months	7 (13.7)	5 (13.9)	0.426		
More than 6 months	12 (23.5)	11 (30.6)	0.605		
**Type of surgical history**					
Abdominal	1 (2.0)	1 (2.9)	0.165		
Orthopedic	2 (3.9)	4 (11.4)	0.502		
Neurosurgery	4 (7.8)	0 (0.0)	0.999		
Cardiovascular	3 (5.9)	11 (31.4)	0.062		
Urological	2 (3.9)	1 (2.9)	0.505		
Lower limb amputation	32 (62.7)	9 (25.7)	0.000	0.003	0.109(0.025-0.478)
Diabetic septic foot debriment	5 (9.8)	8 (22.9)	0.527		
Fasciotomy	1 (2.0)	1 (2.9)	0.933		
Malignancy	1 (2.0)	0 (0.0)	0.999		
**GCS score**					

Severe (≤8)	17 (14.3)	41 (30.6)	0.003	0.242	2.359(0.560-9.936)
Moderate (9 - 12)	60 (50.4)	84 (62.7)	0.050		
Mild (≥13)	42 (35.3)	9 (6.7)	<0.001	0.957	0.957(0.364-2.520)
**Number of comorbidities**	*2.08±1.00*	*2.51±0.83*	<0.001	0.887	0.887(0.481-1.638)
**Type of comorbidities**					
Diabetes Mellitus	97 (81.5)	116 (86.6)	0.273		
Hypertension	93 (78.2)	121 (94.8)	0.009	0.882	1.120(0.251-4.989)
Asthma	2 (1.7)	7 (5.2)	0.149		
Chronic Obstructive Pulmonary Disease	4 (3.4)	1 (0.7)	0.173		
Coronary Artery Disease	15 (12.6)	45 (33.6)	<0.001	0.047	3.128(1.016-9.629)
Congestive Heart Disease	6 (5.0)	4 (3.0)	0.407		
Chronic renal disease	6 (5.0)	5 (3.7)	0.611		
Old Malignancy	2 (1.7)	3 (2.2)	0.751		
Liver diseases	0 (0.0)	2 (1.5)	0.999		
Central Nervous System	22 (18.5)	32 (23.9)	0.297		
**Need for Mechanical Ventilation**	37 (31.1)	112 (83.6)	<0.001	0.464	1.881(0.346-10.220)
**Number of organ failure**	*2.33±1.55*	*4.50±1.30*	<0.001	0.431	1.814(0.412-7.977)
Liver failure	78 (65.5)	112 (83.6)	0.001	0.727	1.355(0.246-7.472)
Acute kidney injury	43 (36.1)	98 (73.1)	<0.001	0.906	1.096(0.240-5.008)
Respiratory failure	40 (33.6)	113 (84.3)	<0.001	0.937	1.098(0.107-11.278)
Central Nervous System	42 (35.3)	105 (78.4)	<0.001	0.728	0.722(0.115-4.535)
Cardiac failure	33 (27.7)	88 (65.7)	<0.001	0.456	1.807(0.381-8.556)
Disseminated intravascular coagulation	34 (28.6)	76 (56.7)	<0.001	0.613	0.613(0.104-3.624)
Venous thromboembolism	6 (5.0)	11 (8.2)	0.320		
**APACHE II score**	*22.76±8.11*	*31.10±6.59*	<0.001	0.026	1.087(1.010-1.170)
**Onset of infection**					

Hospital acquired infection	41 (34.5)	34 (25.4)	0.116		
Community acquired infection	78 (65.5)	100 (74.6)	-		
**Source of infection**					
Respiratory tract infection	41 (34.5)	75 (56.0)	0.001	0.718	1.214(0.425-3.468)
Urinary tract infection	18 (15.1)	27 (20.1)	0.298		
Abdominal infection	2 (1.7)	2 (1.5)	0.905		
Soft tissue/skin infection	43 (36.1)	22 (16.4)	<0.001	0.640	0.640(0.205-1.993)
Surgical sit infection	14 (11.8)	7 (5.2)	0.066		
CNS infection	1 (0.8)	0 (0.0)	0.999		
Unknown	0 (0.0)	1 (0.7)	0.999		
**MDRO**	16 (13.4)	62 (46.3)	<0.001	<0.001	7.318(2.839-18.864)

GCS*: Glasgow Coma Scale.* APACHE II: *Acute Physiology and Chronic Health Evaluation.* CNS: *Central Nervous System.* MDRO: *Multiple Drug Resistant organism.* Hosmer-Lemeshow (p value = 0.658).
